# Why cultural safety rather than cultural competency is required to achieve health equity: a literature review and recommended definition

**DOI:** 10.1186/s12939-019-1082-3

**Published:** 2019-11-14

**Authors:** Elana Curtis, Rhys Jones, David Tipene-Leach, Curtis Walker, Belinda Loring, Sarah-Jane Paine, Papaarangi Reid

**Affiliations:** 1 Te Kupenga Hauora Māori, Faculty of Medical and Health Sciences, University of Auckland, Auckland, New Zealand; 2 Faculty of Education, Humanities and Health Sciences, Eastern Institute of Technology, Napier, New Zealand; 3 Te Kaunihera Rata of Aotearoa, Medical Council of New Zealand, Wellington, New Zealand

**Keywords:** Cultural safety, Cultural competency, Indigenous, Māori, Disparities, Inequity, Ethnic

## Abstract

**Background:**

Eliminating indigenous and ethnic health inequities requires addressing the determinants of health inequities which includes institutionalised racism, and ensuring a health care system that delivers appropriate and equitable care. There is growing recognition of the importance of cultural competency and cultural safety at both individual health practitioner and organisational levels to achieve equitable health care. Some jurisdictions have included cultural competency in health professional licensing legislation, health professional accreditation standards, and pre-service and in-service training programmes. However, there are mixed definitions and understandings of cultural competency and cultural safety, and how best to achieve them.

**Methods:**

A literature review of 59 international articles on the definitions of cultural competency and cultural safety was undertaken. Findings were contextualised to the cultural competency legislation, statements and initiatives present within Aotearoa New Zealand, a national Symposium on Cultural Competence and Māori Health, convened by the Medical Council of New Zealand and Te Ohu Rata o Aotearoa – Māori Medical Practitioners Association (Te ORA) and consultation with Māori medical practitioners via Te ORA.

**Results:**

Health practitioners, healthcare organisations and health systems need to be engaged in working towards cultural safety and critical consciousness. To do this, they must be prepared to critique the ‘taken for granted’ power structures and be prepared to challenge their own culture and cultural systems rather than prioritise becoming ‘competent’ in the cultures of others. The objective of cultural safety activities also needs to be clearly linked to achieving health equity. Healthcare organisations and authorities need to be held accountable for providing culturally safe care, as defined by patients and their communities, and as measured through progress towards achieving health equity.

**Conclusions:**

A move to cultural safety rather than cultural competency is recommended. We propose a definition for cultural safety that we believe to be more fit for purpose in achieving health equity, and clarify the essential principles and practical steps to operationalise this approach in healthcare organisations and workforce development. The unintended consequences of a narrow or limited understanding of cultural competency are discussed, along with recommendations for how a broader conceptualisation of these terms is important.

## Introduction

Internationally, Indigenous and minoritorised ethnic groups experience inequities in their exposure to the determinants of health, access to and through healthcare and receipt of high quality healthcare [1]. The role of health providers and health systems in creating and maintaining these inequities is increasingly under investigation [2]. As such, the cultural competency and cultural safety of healthcare providers are now key areas of concern and issues around how to define these terms have become paramount, particularly within a Aotearoa New Zealand (NZ) context [3]. This article explores international literature to clarify the concepts of cultural competency and cultural safety in order to better inform both local and international contexts.

In NZ, Māori experience significant inequities in health compared to the non-Indigenous population. In 2010–2012, Māori life expectancy at birth was 7.3 years less than non-Māori [4] and Māori have on average the poorest health status of any ethnic group in NZ [5, 6]. Although Māori experience a high level of health care need, Māori receive less access to, and poorer care throughout, the full spectrum of health care services from preventative to tertiary care [7, 8]. This is reflected in lower levels of investigations, interventions, and medicines prescriptions when adjusted for need [8, 9]. Māori are consistently and significantly less likely to: get understandable answers to important questions asked of health professionals; have health conditions explained in understandable terms; or feel listened to by doctors or nurses [10]. The disturbing health and social context for Māori and significant inequities across multiple health and social indicators described above provide the ‘needs-based’ rationale for addressing Māori health inequities [8]. There are equally important ‘rights-based’ imperatives for addressing Indigenous health and health equity [11], that are reinforced by the United Nations Declaration on the Rights of Indigenous Peoples [12] and Te Tiriti o Waitangi (Treaty of Waitangi) in NZ.

There are multiple and complex factors that drive Indigenous and ethnic health inequities including a violent colonial history that resulted in decimation of the Māori population and the appropriation of Māori wealth and power, which in turn has led to Māori now having differential exposure to the determinants of health [13] [14] and inequities in access to health services and the quality of the care received. Framing ethnic health inequities as being predominantly driven by genetic, cultural or biological differences provides a limited platform for in-depth understanding [15, 16]. In addition, whilst socio-economic deprivation is associated with poorer health outcomes, inequities remain even after adjusting for socio-economic deprivation or position [17]. Health professionals and health care organisations are important contributors to racial and ethnic inequities in health care [2, 13]. The therapeutic relationship between a health provider and a patient is especially vulnerable to the influence of intentional or unintentional bias [18, 19] leading to the *“paradox of well-intentioned physicians providing inequitable care* [20]. Equitable care is further compromised by poor communication, a lack of partnership via participatory or shared decision-making, a lack of respect, familiarity or affiliation and an overall lack of trust [18]. Healthcare organisations can influence the structure of the healthcare environment to be less likely to facilitate implicit (and explicit) bias for health providers. Importantly, it is not lack of awareness about ‘the culture of other groups’ that is driving health care inequities - inequities are primarily due to unequal power relationships, unfair distribution of the social determinants of health, marginalisation, biases, unexamined privilege, and institutional racism [13]. Health professional education and health institutions therefore need to address these factors through health professional education and training, organisational policies and practices, as well as broader systemic and structural reform.

Eliminating Indigenous and ethnic health inequities requires addressing the social determinants of health inequities including institutional racism, in addition to ensuring a health care system that delivers appropriate and equitable care. There is growing recognition of the importance of cultural competency and cultural safety at both individual health practitioner and organisational levels to achieve equitable health care delivery. Some jurisdictions have included cultural competency in health professional licensing legislation [21], health professional accreditation standards, and pre-service and in-service training programmes [22–25]. However, there are mixed definitions and understandings of cultural competency and cultural safety, and how best to achieve them. This article reviews how concepts of cultural competency and cultural safety (and related terms such as cultural sensitivity, cultural humility etc) have been interpreted. The unintended consequences of a narrow or limited understanding of cultural competency are discussed, along with recommendations for why broader conceptualisation of these terms is needed to achieve health equity. A move to cultural safety is recommended, with a rationale for why this approach is necessary. We propose a definition for cultural safety and clarify the essential principles of this approach in healthcare organisations and workforce development.

## Methods and positioning

This review was originally conducted to inform the Medical Council of New Zealand, in reviewing and updating its approach to cultural competency requirements for medical practitioners in New Zealand Aotearoa. The review and its recommendations are based on the following methods:

- An international literature review on cultural competency and cultural safety.
- A review of cultural competency legislation, statements and initiatives in NZ, including of the Medical Council of New Zealand (MCNZ).
- Inputs from a national Symposium on Cultural Competence and Māori Health, convened for this purpose by the MCNZ and Te Ohu Rata o Aotearoa – Māori Medical Practitioners Association (Te ORA) [26].
- Consultation with Māori medical practitioners (through Te ORA).

The authors reflect expertise that includes Te ORA membership, membership of the Australasian Leaders in Indigenous Medical Education (LIME) (a network to ensure the quality and effectiveness of teaching and learning of Indigenous health in medical education), medical educationalist expertise and Indigenous medical practitioner and public health medicine expertise across Australia and NZ. This experience has been at the forefront of the development of cultural competency and cultural safety approaches within NZ. The analysis has been informed by the framework of van Ryn and colleagues [27] which frames health provider behaviour within a broader context of societal racism. They note the importance of shifting “*the framing of the problem, from ‘the impact of patient race’ to the more accurate ‘impact of racism’….on clinician cognitions, behaviour, and clinical decision making”* [27].

This review and analysis has been conducted from an Indigenous research positioning that draws from Kaupapa Māori theoretical and research approaches. Therefore, the positioning used to undertake this work aligns to effective Kaupapa Māori research practice that has been described by Curtis (2016) as: transformative; beneficial to Māori; under Māori control; informed by Māori knowledge; aligned with a structural determinants approach to critique issues of power, privilege and racism and promote social justice; non-victim-blaming and rejecting of cultural-deficit theories; emancipatory and supportive of decolonisation; accepting of diverse Māori realities and rejecting of cultural essentialism; an exemplar of excellence; and free to dream [28].

The literature review searched international journal databases and the grey literature. No year limits were applied to the original searching. Databases searched included: Medline, Psychinfo, Cochrane SR, ERIC, CINAHL, Scopus, Proquest, Google Scholar, EbscoHost and grey literature. Search terms included MeSH terms of cultural competence (key words: cultural safety, cultural awareness, cultural competence, cultural diversity, cultural understanding, knowledge, expertise, skill, responsiveness, respect, transcultural, multicultural, cross-cultur*); education (key words: Educat*, Traini*, Program*, Curricul*, Profession*, Course*, Intervention, Session, Workshop, Skill*, Instruc*, program evaluation); Health Provider (key words: provider, practitioner, health professional, physician, doctor, clinician, primary health care, health personnel, health provider, nurse); Health Services Indigenous (key words: health services Indigenous, ethnic* Minorit*, Indigenous people*, native people). A total of 51 articles were identified via the search above and an additional 8 articles were identified via the authors’ opportunistic searching. A total of 59 articles published between 1989 and 2018 were used to inform this review. Articles reviewed were sourced from the USA, Canada, Australia, NZ, Taiwan and Sweden (Additional file 1 Table S1).

In addition to clarifying concepts of cultural competence and cultural safety, a clearer understanding is required of how best to train and monitor for cultural safety within health workforce contexts. An assessment of the availability and effectiveness of tools and strategies to enhance cultural safety is beyond the scope of this review, but is the subject of a subsequent review in process.

## Reviewing cultural competency

Cultural competency is a broad concept that has various definitions drawing from multiple frameworks. Overall, this concept has varying interpretations within and between countries (see Table 1 for specific examples). Introduced in the 1980s, cultural competency has been described as a recognised approach to improving the provision of healthcare to ethnic minority groups with the aim of reducing ethnic health disparities [31].

**Table 1:** Definitions and Concepts of Related Terms

Terms	Definition/Concept Examples	Sources
Culture	The integrated pattern of human behaviour that includes thoughts, communications, actions, customs, beliefs, values, and institutions of a racial, ethnic, religious, or social group.	[29]
	The accumulated socially acquired result of shared geography, time, ideas and human experience. Culture may or may not involve kinship, but meanings and understandings are collectively held by group members. Culture is dynamic and mobile and changes according to time, individuals and groups.	[30]
	There is a tendency within healthcare to equate culture with essentialized notions of race and ethnicity, which can lead to practices that separate culture from its social, economic and political context. Narrow conceptualizations of culture and identity may limit the effectiveness of particular approaches, and a focus on specific cultural information may inadvertently promote stereotyping.	[31]
Cultural Awareness	a beginning step towards understanding that there is difference. Many people undergo courses designed to sensitise them to formal ritual rather than the emotional, social, economic and political context in which people exist	[30]
	[is] concerned with having knowledge about cultural but, more specifically, ethnic diversity.	[32]
	an individual’s awareness of her/his own views such as ethnocentric, biased and prejudiced beliefs towards other cultures (p. e120)	[33]
	essentially the basic acknowledgment of differences between cultures	[34]
	recognizing that there are differences between cultures	[35]
Cultural Sensitivity	alerts students to the legitimacy of difference and begins a process of self-exploration as the powerful bearers of their own life experience and realities and the impact this may have on others.	[30]
	building on the awareness of difference through cultural acceptance, respect and understanding	[36]
	builds on cultural awareness’ acknowledgment of difference with the addition of the requirement of respecting other cultures	[34]
	where students start to analyse their own realities and the impact that this may have on others.	[35]
Cultural Humility	incorporates a lifelong commitment to self-evaluation and self-critique, to redressing the power imbalances in the patient-physician dynamic, and to developing mutually beneficial and non-paternalistic clinical and advocacy partnerships with communities on behalf of individuals and defined populations.	[37]
	entails valuing life-long learning and critical self-reflection, along with a respectful and inquisitive approach whereby practitioners are expected to seek knowledge from their clients regarding their cultural and structural influences rather than assuming understanding or expertise about a culture outside of their own	[38]
	defined as having an interpersonal stance that is other-oriented rather than self-focused, characterized by respect and lack of superiority toward an individual’s cultural background and experience.	[39]
	does not have an endpoint or goal; there is no objective of mastering another culture. Rather it is a continual process of self-reflection and self-critique that overtly addresses power inequities between providers and clients. Attaining cultural humility becomes not a goal but an active process, an ongoing way of being in the world and being in relationships with others and self.	[40]
Cultural Security	it legitimises and values cultural differences to ensure no harm is caused and ultimately links understandings and actions	[41]
	seeks to create interactions between health workers and health service users that do ‘not compromise the legitimate cultural rights, views, values and expectations of Aboriginal people’	[42]
Cultural Respect	The ‘Cultural Respect Framework for Aboriginal and Torres Strait Islander Health 2004–2009’ identifies the goal of cultural respect as ‘uphold[ing] the rights of Aboriginal and Torres Strait Islander peoples to maintain, protect and develop their culture and achieve equitable health outcomes	[42]
	The model entails four basic elements highlighting the importance of ‘Knowledge and Awareness’ that informs ‘Skilled Practice and Behaviours’ as well as the development of ‘Strong Relationships’ between (health) institutions, individuals and communities in order to achieve an ‘Equity of Outcomes’	[43]
Cultural Adaptation	all modifications made to standard service methods in order to make services more acceptable, relevant, useful, and/or effective for diverse populations	[44]
Transcultural Competence	the ability to interact with clientele who come from a range of different cultural backgrounds	[43]
Transcultural Effectiveness	ability of organisations and systems ‘to acknowledge and respond to unique and diverse perspectives and support non- discriminatory practice’ (p. S51). These authors maintain, furthermore, that transculturally competent practitioners should recognise organisational and systemic obstacles and actively seek ways to modify them	[43]
Transcultural Nursing	Leininger (1994) defined transcultural nursing as being a subfield of nursing, focusing on comparative study and analysis of different cultures and is the basis on which the theory of culture care diversity and universality was formed. The goal of transcultural nurses being to “identify, test, understand and use a body of transcultural nursing knowledge and practices which is culturally derived in order to provide culturally specific nursing care to people”	[45]
Culturally Unsafe	“any actions that diminish, demean or disempower the cultural identity and well-being of an individual’	[46, 47]
	any action that diminishes, demeans, or disempowers the cultural identity and well-being of an individual	[48]
Cultural Destructiveness	attitudes, policies, and practices that are destructive to cultures and consequently to the individuals within the culture such as cultural genocide	[29]
Cultural Incapacity	system or agencies that lack the capacity to help minority clients or communities due to extreme bias, paternalism and a belief in the racial superiority of the dominant group	[29]
Cultural Blindness	system or agencies that function with the belief that colour or culture make no difference and that all people are the same	[29]
Cultural Pre-competence	an agency that realises its weaknesses in serving minorities and attempts to improve some aspect of their services to a specific population	[29]
Cultural Proficiency	agencies that hold culture in high esteem, who seek to add to the knowledge base of culturally competent practice by conducting research and developing new therapeutic approaches based on culture	[29]

One of the earliest [49] and most commonly cited definitions of cultural competency is sourced from a 1989 report authored by Cross and colleagues in the United States of America [29] (p.13):

*Cultural competence is a set of congruent behaviours, attitudes, and policies that come together in a system, agency, or among professionals and enable that system, agency, or those professionals to work effectively in cross-cultural situations.*

Cross et al. [29] contextualized cultural competency as part of a continuum ranging from the most negative end of *cultural destructiveness* (e.g. attitudes, policies, and practices that are destructive to cultures and consequently to the individuals within the culture such as cultural genocide) to the most positive end of *cultural proficiency* (e.g. agencies that hold culture in high esteem, who seek to add to the knowledge base of culturally competent practice by conducting research and developing new therapeutic approaches based on culture). Other points along this continuum include: *cultural incapacity*, *cultural blindness* and *cultural pre-competence* (Table 1).

By the time that cultural competency became to be better understood in the late 1990s, there had been substantial growth in the number of definitions, conceptual frameworks and related terms [31, 50–52]. Table 1 provides a summary of the multiple, interchangeable, terms such as: *cultural awareness*; *cultural sensitivity*; *cultural humility*; *cultural security*; *cultural respect*; *cultural adaptation*; and *transcultural competence* or *effectiveness*. Unfortunately, this rapid growth in terminology and theoretical positioning(s), further confused by variations in policy uptake across the health sector, reduced the potential for a common, shared understanding of what cultural competency represents and therefore what interventions are required. Table 2 outlines the various definitions of cultural competency from the literature.

**Table 2:** Key Definitions and Concepts of Cultural Competency

Term	Definition/Concept Examples	Sources
Cultural Competency	a set of congruent behaviours, attitudes, and policies that come together in a system, agency, or among professionals and enable that system, agency, or those professionals to work effectively in cross-cultural situations.	[29]
	acknowledges and incorporates - at all levels - the importance of culture, the assessment of cross-cultural relations, vigilance towards the dynamics that result from cultural differences, the expansion of cultural knowledge, and the adaptation of services to meet culturally-unique needs.	[29]
	a culturally competent counsellor must acquire ‘awareness, knowledge, and skills needed to function effectively in a pluralistic democratic society’, i.e., develop the ‘ability to communicate, interact, negotiate, and intervene on behalf of clients from diverse backgrounds	[43]
	the ability of individuals to establish effective interpersonal and working relationships that supersede cultural differences by recognizing the importance of social and cultural influences on patients, considering how these factors interact, and devising interventions that take these issues into account	[53]
	the ability of systems to provide care to patients with diverse values, beliefs and behaviours, including tailoring delivery to meet patients’ social, cultural, and linguistic needs	[54]
	a more comprehensive, skill-based concept that purposefully involves the system in addition to the patients and has been conceived of as an on-going quality improvement process, relevant across individual, organisational, systemic and professional levels	[50]
	a complex know-act grounded in critical reflection and action, which the health care professional draws upon to provide culturally safe, congruent, and effective care in partnership with individuals, families, and communities living health experiences, and which takes into account the social and political dimensions of care	[55]
	the ability to work and communicate effectively and appropriately with people from culturally different backgrounds. While appropriateness implies not violating the valued rules, effectiveness means achieving the valued goals and outcomes in intercultural interactions	[33]
	the ability to acknowledge, appreciate, and respect the values, preferences, and expressed needs of clients. Cultural competence is also the ability to resolve differences and identify solutions that reduce interference from various cultural factors…This article defines cultural competency as encompassing open-mindedness and a respect for people, families, and societies of various cultural backgrounds. Being able to translate this cultural knowledge and these skills into practice may help enhance the cultural appropriateness of healthcare.	[51]
	the development of a skill set for more effective provider-patient communication. They stressed the importance of providers’ understanding the relationship between cultural beliefs and behaviour and developing skills to improve quality of care to diverse populations. Several informants expressed concern about the persistence of stereotypic teaching strategies (such as treating Hispanics one way and African Americans another). They mentioned additional components that were underemphasized such as empathy, exploring socioeconomic issues, and addressing bias in the clinical encounter	[56]
	Social workers describe cultural competence as a continual process of striving to become increasingly self-aware, to value diversity, and to become knowledgeable about cultural strengths	[57]
	frequently approached in ways which limit its goals to knowledge of characteristics, cultural beliefs, and practices of different non-majority groups, and skills and attitudes of empathy and compassion in interviewing and communicating with non-majority groups. Achieving cultural competence is thus often viewed as a static outcome: One is “competent” in interacting with patients from diverse backgrounds much in the same way as one is competent in performing a physical exam or reading an EKG.	[58]
	an awareness of cultural diversity and the ability to function effectively, and respectfully, when working with and treating people of different cultural backgrounds. Cultural competence means a doctor has the attitudes, skills and knowledge needed to achieve this. A culturally competent doctor will acknowledge: That New Zealand has a culturally diverse population. That a doctor’s culture and belief systems influence his or her interactions with patients and accepts this may impact on the doctor-patient relationship. That a positive patient outcome is achieved when a doctor and patient have mutual respect and understanding	[23]
	At the level of the individual practitioner, cultural competence enables increased awareness and understanding of the perspectives and lived realities of Māori clients, which in turn facilitates genuine engagement, trust, and information sharing contributing to enhanced clinical outcomes.	[59]
Organisational Cultural Competency	To achieve organizational cultural competence within the health care leadership and workforce, it is important to maximize diversity. This may be accomplished through: Establishing programs for minority health care leadership development and strengthening existing programs. The desired result is a core of professionals who may assume influential positions in academia, government, and private industry. Hiring and promoting minorities in the health care workforce. Involving community representatives in the health care organization’s planning and quality improvement meetings.	[54]
	Organisational cultural competence involves strategies that maximise diversity and incorporate leadership and workforce issues. Specifically, ethnic matching and working with communities. The lack of diversity in health care leadership and the workforce has been identified as a barrier to culturally competent care, and studies have shown that health care quality and racial and ethnic diversity are linked	[60]
Systemic Cultural Competency	To achieve systemic cultural competence (e.g., in the structures of the health care system) it is essential to address such initiatives as conducting community assessments, developing mechanisms for community and patient feedback, implementing systems for patient racial/ethnic and language preference data collection, developing quality measures for diverse patient populations, and ensuring culturally and linguistically appropriate health education materials and health promotion and disease prevention interventions.	[54]
	At the system level, the structures of the health care system are attended to and include strategies, such as ethnicity data collection and strategic planning. Ethnicity data collection can assist in the planning of improvements to services by comparing access to services and outcomes of care.	[60]

Cultural competence was often defined within an individually-focused framework, for example, as:

*the ability of individuals to establish effective interpersonal and working relationships that supersede cultural differences by recognizing the importance of social and cultural influences on patients, considering how these factors interact, and devising interventions that take these issues into account* [53] (p.2).

Some positionings for cultural competency have been critiqued for promoting the notion that health-care professionals should strive to (or even can) master a certain level of functioning, knowledge and understanding of Indigenous culture [61]. Cultural competency is limited when it focuses on acquiring knowledge, skills and attitudes as this infers that it is a ‘static’ level of achievement [58]:

*“cultural competency” is frequently approached in ways which limit its goals to* ***knowledge*** *of characteristics, cultural beliefs, and practices of different nonmajority groups, and* ***skills*** *and* ***attitudes*** *of empathy and compassion in interviewing and communicating with nonmajority groups. Achieving cultural competence is thus often viewed as a static outcome: One is “competent” in interacting with patients from diverse backgrounds much in the same way as one is competent in performing a physical exam or reading an EKG.* ***Cultural competency is not an abdominal exam****. It is not a static requirement to be checked off some list but is something beyond the somewhat rigid categories of knowledge, skills, and attitudes (*p.783).

By the early 2000s, governmental policies and cultural competency experts [50, 54] had begun to articulate cultural competency in terms of both individual and organizational interventions, and describe it with a broader, systems-level focus, e.g.:

*the ability of systems to provide care to patients with diverse values, beliefs and behaviours, including tailoring delivery to meet patients’ social, cultural, and linguistic needs* [54] (p. v).

Moreover, some commentators began to articulate the importance of critical reflection to cultural competency. For example, Garneau and Pepin [55] align themselves more closely to the notion of *cultural safety* when they describe cultural competency as:

*a complex know-act grounded in critical reflection and action, which the health care professional draws upon to provide culturally safe, congruent, and effective care in partnership with individuals, families, and communities living health experiences, and which takes into account the social and political dimensions of care* [55] (p. 12).

## Reviewing cultural safety

A key difference between the concepts of cultural competency and cultural safety is the notion of ‘power’. There is a large body of work, developed over many years, describing the nuances of the two terms [34, 36, 38, 43, 46, 49, 59, 62–69]. Similar to cultural competency, this concept has varying interpretations within and between countries. Table 3 summarises the definitions and use of cultural safety from the literature. Cultural safety foregrounds power differentials within society, the requirement for health professionals to reflect on interpersonal power differences (their own and that of the patient), and how the transfer of power within multiple contexts can facilitate appropriate care for Indigenous people and arguably for all patients [32].

**Table 3:** Key Definitions and Concepts of Cultural Safety

Term	Definition/Concept Examples	Sources
Cultural Safety	is an outcome of nursing and midwifery education that enables safe service to be defined by those that receive the service	[30]
	a focus for the delivery of quality care through changes in thinking about power relationships and patients’ rights	[32]
	The skill for nurses and midwives does not lie in knowing the customs of ethnospecific cultures. Rather, cultural safety places an obligation on the nurse or midwife to provide care within the framework of recognizing and respecting the difference of any individual. But it is not the nurse or midwife who determines the issue of safety. It is consumers or patients who decide whether they feel safe with the care that has been given	[32]
	The focus of cultural safety teaching is to educate student nurses and student midwives: - to examine their own realities and the attitudes they bring to each new person they encounter in their practice; − to be open minded and flexible in their attitudes toward people who are different from themselves, to whom they offer or delivers - not to blame the victims of historical and social processes for their current plight; − to produce a workforce of well educated, self-aware registered nurses and midwives who are culturally safe to practice	[32]
	where there is no inadvertent disempowering of the recipient, indeed where recipients are involved in the decision making and become part of a team effort to maximise the effectiveness of the care. The model pursues more effective practice through being aware of difference, decolonising, considering power relationships, implementing reflective practice, and by allowing the patient to determine what safety means.	[65]
	a nurse who could objectively evaluate his or her own culture and be familiar about the theory of power structures, is also a culturally safe nurse in all contexts	[35]
	places an emphasis on the health worker understanding their own culture and identity, and how this manifests in their practice. Thus, cultural safety is concerned with both systemic and individual change with the aim of examining processes of identity formation and enhancing health workers’ awareness of their own identity and its impact on the care they provide to people from indigenous cultural groups.	[42]
	aims to directly address the effects of colonialism within the dominant health system by focusing on the level of cultural safety felt by an individual seeking health care. The responsibility to recognize and protect a person’s cultural identity (and hence maintain their cultural safety) lies with the health service. Emphasis is placed on assisting the health worker to understand processes of identity and culture, and how power imbalances or relationships can be culturally unsafe (and thus, detrimental to a person’s health and wellbeing)	[42]
	a strategic and intensely practical plan to change the way healthcare is delivered to Aboriginal people. In particular, the concept is used to express an approach to healthcare that recognizes the contemporary conditions of Aboriginal people which result from their post-contact history.	[63]
	the movement from cultural competence to cultural safety is not merely another step on a linear continuum, but rather a more dramatic change of approach. This conceptualization of cultural safety represents a more radical, politicized understanding of cultural consideration, effectively rejecting the more limited culturally competent approach for one based not on knowledge but rather on power.	[63]
	best nurtured in conjunction with other embedded philosophies such as decolonization, symbolic interactionism, understanding social interaction in context, and the social justice imperative to avoid further harm from domination and oppression	[47] (
	a constant self-evaluation by a provider to ensure they’re focusing on the individual and are not being influenced by assumptions about that individual’s cultural background or social or economic status…also helps alter the colonial relationship and makes safe space for Indigenous peoples within the system and thereby allowing them to help reshape the system itself	[62]
	provides for the formal recognition of power relations within health care (and in particular nursing) interactions. By adopting cultural safety it becomes not only possible but inevitable that an exploration of the assumptions underlying practice, brought by both individuals and the profession will occur. This reflective model is effective on the individual, institutional and professional levels, and encourages identification of the assumptions and preconceptions that structure practice	[66]
	a powerful means of conveying the idea that cultural factors critically influence the relationship between carer and patient. Cultural safety focuses on the potential differences between health providers and patients that have an impact on care and aims to minimize any assault on the patient’s cultural identity. Specifically, the objectives of cultural safety in nursing and midwifery training are to educate students to examine their own realities and attitudes they bring to clinical care, to educate them to be open-minded towards people who are different from themselves, to educate them not to blame the victims of historical and social processes for their current plight, and to produce a workforce of well-educated and self-aware health professionals who are culturally safe to practice as defined by the people they serve.	[46]
	does not emphasize developing “competence” through knowledge about the cultures with which professionals are working. Instead, cultural safety emphasizes recognizing the social, historical, political and economic circumstances that create power differences and inequalities in health and the clinical encounter	[46]
	is underpinned by a social justice framework and requires individuals to undertake a process of personal reflection. Cultural safety is therefore a holistic and shared approach, where all individuals feel safe, can undertake learning [36] together with dignity, and demonstrate deep listening	[36]
	is grounded in critical theoretical perspectives and draws attention to critically oriented knowledge, such as racialization, culturalism, institutional racism and discrimination, and health and health care inequities	[68]
	extends beyond cultural awareness, sensitivity, and skills-based competencies and is predicated on understanding the power differentials inherent in health care service delivery to redress these inequities through educational processes, focusing on reflexive thinking	[70]
	is informed philosophically by “emancipatory or neocolonisation theoretical perspectives” and by an emphasis on social justice. Grounded in critical theory, cultural safety invites the nurse	[71]
	goes beyond describing the practices of other ethnic groups, because such a strategy can lead to a checklist mentality that essentialises group members. Furthermore, a nurse having knowledge of a client’s culture could be disempowering for a client who is disenfranchised from their own culture, and could be seen as the continuation of a colonising process that is both demeaning and disempowering or appropriating. Culturally safe nurses focus on self-understanding and the emphasis is on what attitudes and values nurses bring to their practice. A key tenet is that ‘a nurse or midwife who can understand his or her own culture and the theory of power relations can be culturally safe in any context’	[60]
	advocates that both professionals and institutions work to establish a safe place for patients, which is sensitive and responsive to their social, political, linguistic, economic and spiritual concerns. Cultural safety is more than an understanding of a patient’s ethnic background as it requires the ‘health professional to reflect on their own cultural identity and on their relative power as a health provider’	[72]
	The curriculum staircase or poutama assumes that students begin their cultural safety education at the bottom of the staircase where they bring with them their personal experiences, knowledge, and biases. Over the next 3 years of their training, the students are assessed on their ability to move to each step. This training focuses on racism awareness, the Treaty of Waitangi, ngā mea Māori (concepts important to Māori), and strategies for institutional change. Hence, the educational process involves movement from awareness to sensitivity, and ultimately to safety.	[48]
	we envisioned that cultural safety might assist nurses to examine how popularized notions of culture and cultural differences are taken up; to develop greater awareness of how individual and societal assumptions and stereotypes operate in practice; and to better recognize how organizational and structural inequities and wider social discourses – within health care and in our society – inevitably influence nurses’ interpretive perspectives and practices.	[73]
Critical Consciousness	If we try to move beyond cultural competency and instead focus on the development of this critical consciousness, what is its object of knowledge? In other words, “What stuff should we learn?” The object of knowledge is not just a series of lists of cultural attributes (which can quickly degrade into dehumanizing stereotypes), nor is it a skill set of questions and demeanours we should assume when encountering a patient who is not like us. We propose that the object of knowledge of these educational efforts is the development of critical consciousness itself, that is, the knowledge and awareness to carry out the social roles and responsibilities of a physician. This way of knowing is a different type of knowledge than that required when studying the biomedical sciences— complementary, but different all the same.	[58]
	Cultural competency is not an abdominal exam. It is not a static requirement to be checked off some list but is something beyond the somewhat rigid categories of knowledge, skills, and attitudes: the continuous critical refinement and fostering of a type of thinking and knowing—a critical consciousness— of self, others, and the world.	[58]

The term cultural safety first was first proposed by Dr. Irihapeti Ramsden and Māori nurses in the 1990s [74], and in 1992 the Nursing Council of New Zealand made cultural safety a requirement for nursing and midwifery education [32]. Cultural safety was described as providing:

*a focus for the delivery of quality care through changes in thinking about power relationships and patients’ rights* [32]*.* (p.493).

Cultural safety is about acknowledging the barriers to clinical effectiveness arising from the inherent power imbalance between provider and patient [65]. This concept rejects the notion that health providers should focus on learning cultural customs of different ethnic groups. Instead, cultural safety seeks to achieve better care through being aware of difference, decolonising, considering power relationships, implementing reflective practice, and by allowing the patient to determine whether a clinical encounter is safe [32, 65]*.*

Cultural safety requires health practitioners to examine themselves and the potential impact of their own culture on clinical interactions. This requires health providers to question their own biases, attitudes, assumptions, stereotypes and prejudices that may be contributing to a lower quality of healthcare for some patients. In contrast to cultural competency, the focus of cultural safety moves to the culture of the clinician or the clinical environment rather than the culture of the ‘exotic other’ patient.

There is debate over whether cultural safety reflects an end point along a continuum of cultural competency development, or, whether cultural safety requires a paradigm shift associated with a transformational jump in cultural awareness. Dr. Irihapeti Ramsden [75] originally described the process towards achieving cultural safety in nursing and midwifery practice as a step-wise progression from cultural awareness through to cultural sensitivity and on to cultural safety. However, Ramsden was clear that the terms cultural awareness and cultural sensitivity were separate concepts and that they were not interchangeable with cultural safety. Despite some authors interpreting Ramsden’s original description of cultural safety as involving three steps along a continuum [35] other authors view a move to cultural safety as more of a ‘paradigm shift’ [63]:

*where the movement from cultural competence to cultural safety is not merely another step on a linear continuum, but rather a more dramatic change of approach. This conceptualization of cultural safety represents a more radical, politicized understanding of cultural consideration, effectively rejecting the more limited culturally competent approach for one based not on knowledge but rather on power* [63]. (p.10).

Regardless of whether cultural safety represents movement along a continuum or a paradigm shift, commentators are clear that the concept of cultural safety aligns with critical theory, where health providers are invited to “examine sources of repression, social domination, and structural variables such as class and power” [71] (p.144) and “social justice, equity and respect” [76] (p.1). This requires a movement to critical consciousness, involving critical self-reflection: “*a stepping back to understand one’s own assumptions, biases, and values, and a shifting of one’s gaze from self to others and conditions of injustice in the world.”* [58] (p.783).

## Why a narrow understanding of cultural competency may be harmful

Unfortunately, regulatory and educational health organisations have tended to frame their understanding of cultural competency towards individualised rather than organisational/systemic processes, and on the acquisition of cultural-knowledge rather than reflective self-assessment of power, priviledge and biases. There are a number of reasons why this approach can be harmful and undermine progress on reducing health inequities.

Individual-level focused positionings for cultural competency perpetuate a process of “othering”, that identifies those that are thought to be different from oneself or the dominant culture. The consequences for persons who experience othering include alienation, marginalization, decreased opportunities, internalized oppression, and exclusion [77]. To foster safe and effective health care interactions, those in power must actively seek to unmask othering practices [78].

“Other-focused” approaches to cultural competency promote oversimplified understandings of other cultures based on cultural stereotypes, including a tendency to homogenise Indigenous people into a collective ‘they’ [79]. This type of cultural essentialism not only leads to health care providers making erroneous assumptions about individual patients which may undermine the provision of good quality care [31, 53, 58, 63, 64], but also reinforces a racialised, binary discourse, used to repeatedly dislocate and destabilise Indigenous identity formations [80]. By ignoring power, narrow approaches to cultural competency perpetuate deficit discourses that place responsibility for problems with the affected individuals or communities [81], overlooking the role of the health professional, the health care system and broader socio-economic structures. Inequities in access to the social determinants of health have their foundations in colonial histories and subsequent imbalances in power that have consistently benefited some over others. Health equity simply cannot be achieved without acknowledging and addressing differential power, in the healthcare interaction, and in the broader health system and social structures (including in decision making and resource allocation) [82].

An approach to cultural competency that focuses on acquiring knowledge, skills and attitudes is problematic because it suggests that competency can be fully achieved through this static process [58]. Cultural competency does not have an endpoint, and a “tick-box” approach may well lull practitioners into a falsely confident space. These dangers underscore the importance of framing cultural safety as an ongoing and reflective process, focused on ‘critical consciousness’. There will still be a need for health professionals to have a degree of knowledge and understanding of other cultures, but this should not be confused with or presented as efforts to address cultural safety. Indeed, as discussed above, this information alone can be dangerous without deep self-reflection about how power and privilege have been redistributed during those processes and the implications for our systems and practice.

By neglecting the organisational/systemic drivers of health care inequities, individual-level focused positionings for cultural competency are fundementally limited in their ability to impact on health inequities. Healthcare organisations influence health provider bias through the structure of the healthcare environment, including factors such as their commitment to workforce training, accountability for equity, workplace stressors, and diversity in workforce and governance [27]. Working towards cultural safety should not be viewed as an intervention purely at the level of the health professional – although a critically conscious and empathetic health professional is certainly important. The evidence clearly emphasises the important role that healthcare organisations (and society at large) can have in the creation of culturally safe environments [31, 32, 46, 60, 69]. Cultural safety initiatives therefore should target both individual health professionals and health professional organisations to intervene positively towards achieving health equity.

Perhaps not surprisingly, the concept of cultural safety is often more confronting and challenging for health institutions, professionals, and students than that of cultural competency. Regardless, it has become increasingly clear that health practitioners, healthcare organisations and health systems all need to be engaged in working towards cultural safety and critical consciousness. To do this, they must be prepared to critique the ‘taken for granted’ power structures and be prepared to challenge their own culture, biases, privilege and power rather than attempt to become ‘competent’ in the cultures of others.

## Redefining cultural safety to achieve health equity

It is clear from reviewing the current evidence associated with cultural competency and cultural safety that a shift in approach is required. We recommend an approach to cultural safety that encompasses the following core principles:

- Be clearly focused on achieving health equity, with measureable progress towards this endpoint;
- Be centred on clarified concepts of *cultural safety* and *critical consciousness* rather than narrow based notions of *cultural competency;*
- Be focused on the application of *cultural safety* within a healthcare *systemic/organizational* context in addition to the *individual* health provider-patient interface;
- Focus on *cultural safety* activities that extend beyond acquiring knowledge about ‘other cultures’ and developing appropriate skills and attitudes and move to interventions that acknowledge and address biases and stereotypes;
- Promote the framing of *cultural safety* as requiring a focus on power relationships and inequities within health care interactions that reflect historical and social dynamics.
- Not be limited to formal training curricula but be aligned across all training/practice environments, systems, structures, and policies.

We recommend that the following definition for cultural safety is adopted by healthcare organisations:

“Cultural safety requires healthcare professionals and their associated healthcare organisations to examine themselves and the potential impact of their own culture on clinical interactions and healthcare service delivery. This requires individual healthcare professionals and healthcare organisations to acknowledge and address their own biases, attitudes, assumptions, stereotypes, prejudices, structures and characteristics that may affect the quality of care provided. In doing so, cultural safety encompasses a critical consciousness where healthcare professionals and healthcare organisations engage in ongoing self-reflection and self-awareness and hold themselves accountable for providing culturally safe care, as defined by the patient and their communities, and as measured through progress towards acheiveing health equity. Cultural safety requires healthcare professionals and their associated healthcare organisations to influence healthcare to reduce bias and achieve equity within the workforce and working environment”.

In operationalising this approach to cultural safety, organisations (health professional training bodies, healthcare organisations etc) should begin with a self-review of the extent to which they meet expectations of *cultural safety* at a systemic and organizational level and identify an action plan for development. The following steps should also be considered by healthcare organisations and regulators to take a more comprehensive approach to cultural safety:

- Mandate evidence of engagement and transformation in *cultural safety* activities as a part of vocational training and professional development;
- Include evidence of cultural safety (of organisations and practitioners) as a requirement for accreditation and ongoing certification;
- Ensure that cultural safety is assessed by the systematic monitoring and assessment of inequities (in health workforce and health outcomes);
- Require *cultural safety* training and performance monitoring for staff, supervisors and assessors;
- Acknowledge that *cultural safety* is an independent requirement that relates to, but is not restricted to, expectations for competency in ethnic or Indigenous health.

## Conclusion

Cultural competency, cultural safety and related terms have been variably defined and applied. Unfortunately, regulatory and educational health organisations have tended to frame their understanding of cultural competency towards individualised rather than organisational/systemic processes, and on the acquisition of cultural-knowledge rather than reflective self-assessment of power, priviledge and biases. This positioning has limited the impact on improving health inequities. A shift is required to an approach based on a transformative concept of cultural safety, which involves a critique of power imbalances and critical self-reflection.

Health practitioners, healthcare organisations and health systems need to be engaged in working towards cultural safety and critical consciousness. To do this, they must be prepared to critique the ‘taken for granted’ power structures and be prepared to challenge their own culture and cultural systems rather than prioritise becoming ‘competent’ in the cultures of others. The objective of cultural safety activities also needs to be clearly linked to achieving health equity. Healthcare organisations and authorities need to be held accountable for providing culturally safe care, as defined by patients and their communities, and as measured through progress towards achieving health equity.

We propose principles and a definition for cultural safety that addresses the key factors identified as being responsible for ethnic inequities in health care, and which we therefore believe is fit for purpose in Aotearoa New Zealand and internationally. We hope this will be a useful starting point for users to further reflect on the work required for themselves, and their organisations, to contribute to the creation of culturally safe environments and therefore to the elimination of Indigenous and ethnic health inequities. More work is needed on how best to train and monitor for cultural safety within health workforce contexts.

## Supplementary information

**Additional file 1: Table S1.** Summary of evidence sources identified from the literature review.

## Data Availability

Not applicable.

## Abbreviations

LIME: Leaders in Indigenous Medical Education network
MCNZ: Medical Council of New Zealand
NZ: Aotearoa New Zealand
Te ORA: Te Ohu Rata o Aotearoa – Māori Medical Practitioners Association
