# Supplementary material for: Why cultural safety rather than cultural competency is required to achieve health equity: a literature review and recommended definition
Source: Int J Equity Health. 2019 Nov 14;18:174. doi: 10.1186/s12939-019-1082-3 (PMC6857221; doi:10.1186/s12939-019-1082-3)
Supplement: Supplementary file 1 — Additional file 1: Table S1. Summary of evidence sources identified from the literature review. [file 12939_2019_1082_MOESM1_ESM.docx]

### Supplemetary Table: Evidence sources identified from the literature review

|  | Author(s) and Title | Year | Country | Type of article |
| --- | --- | --- | --- | --- |
| 1 | **Alizadeh S, Chavan M.** Cultural competence dimensions and outcomes: a systematic review of the literature. Health & Social Care in the Community 24(6): e117-e130. | 2016 | Australia | Systematic review |
| 2 | **Beach MC, et al.** Cultural competence: A systematic review of health care provider educational interventions. Medical Care 43(4): 356-373. | 2005 | USA | Literature review, 34 studies included. |
| 3 | **Betancourt JR, Greean AR, Carillo JE.** Cultural Competence in Health Care: Emerging Frameworks and Practical Approaches. The Commonwealth Fund. | 2002 | USA | Primary research |
| 4 | **Betancourt JR, et al.** Defining Cultural Competence: A Practical Framework for Addressing Racial/Ethnic Disparities in Health and Health Care. Public Health Reports 118(4): 293-302. | 2003 | USA | Literature review |
| 5 | **Betancourt JR, et al.** Cultural competence and health care disparities: key perspectives and trends. Health Affairs 24(2): 499-505. | 2005 | USA | Key informant interviews |
| 6 | **Blanchet Garneau A.** Applying cultural safety beyond Indigenous contexts: Insights from health research with Amish and Low German Mennonites. Nursing Inquiry 25(1). | 2018 | Canada | Perspective/viewpoint article |
| 7 | **Blanchet Garneau A, Pepin J.** Cultural competence: a constructivist definition. Journal of Transcultural Nursing 26(1): 9-15. | 2015 | Canada | Literature review |
| 8 | **Blanchet-Cohen N, Richardson/Kinewesquao C.** Foreword: fostering cultural safety across contexts. AlterNative: An International Journal of Indigenous Peoples 13(3): 138-141. | 2017 | Canada | Editorial |
| 9 | **Brascoupé S, Waters C.** Cultural Safety: Exploring the Applicability of the Concept of Cultural Safety to Aboriginal Health and Community Wellness. Journal of Aboriginal Health 5(2): 6-41. | 2009 | Canada | Literature review and case-studies |
| 10 | **Browne AJ, et al.** Cultural safety and the challenges of translating critically oriented knowledge in practice. Nursing Philosophy 10(3): 167-179. | 2009 | Canada | Knowledge-translation study (using cultural safety in practice) |
| 11 | **Chang ES, et al.** Integrating cultural humility into health care professional education and training. Advances in Health Sciences Education 17(2): 269-278. | 2012 | USA | Perspective/viewpoint article |
| 12 | **Clifford A, et al.** Interventions to improve cultural competency in health care for Indigenous peoples of Australia, New Zealand, Canada and the USA: a systematic review. International Journal for Quality in Health Care 27(2): 89-98. | 2015 | Australia | Literature Review (16 articles) |
| 13 | **Cross TL, Bazron BJ, lsaccs MR.** Towards a Culturally Competent System of Care: A Monograph on Effective Services for Minority Children Who Are Severely Emotionally Disturbed. Washington DC: CASSP Technical Assistance Centre, Georgetown University Child Development Center. | 1989 | USA | Report |
| 14 | **Darroch F, et al.** The United States Does CAIR About Cultural Safety: Examining Cultural Safety Within Indigenous Health Contexts in Canada and the United States. Journal of Transcultural Nursing 28(3): 269-277. | 2017 | USA | Policy/literature review in USA and Canada |
| 15 | **Dell EM, et al.** Cultural Safety and Providing Care to Aboriginal patients in the Emergency Department. CJEM Canadian Journal of Emergency Medical Care 18(4): 301-305. | 2016 | Canada | Perspective/viewpoint article |
| 16 | **DeSouza R.** Wellness for all: The possibilities of cultural safety and cultural competence in New Zealand. Journal of Research in Nursing 13(2): 125-135. | 2008 | New Zealand | Perspective/viewpoint article |
| 17 | **Doutrich D, et al.** Cultural Safety in New Zealand and the United States: Looking at a Way Forward Together. Journal of Transcultural Nursing 23(2): 143-150. | 2012 | USA | Key informant interviews |
| 18 | **Downing R, et al.** Indigenous cultural training for health workers in Australia. International Journal for Quality in Health Care 23(3): 247-257. | 2011 | Australia | Literature review |
| 19 | **Duke J, Connor M, Mceldowney R.**Becoming a culturally competent health practitioner in the delivery of culturally safe care: A process oriented approach. Journal of cultural diversity 16(2):40-49. | 2009 | New Zealand | Perspective/viewpoint article |
| 20 | **Eriksson C, Eriksson L.**  Inequities in health care: lessons from New Zealand : A qualitative interview study about the cultural safety theory. Thesis. The Red Cross University College in Stockholm, Sweden. | 2017 | New Zealand/Sweden | Primary qualitative research |
| 21 | **Fleming T, et al.** Impact of a continuing professional development intervention on midwifery academics' awareness of cultural safety. Women And Birth: Journal Of The Australian College Of Midwives 30(3): 245-252. | 2017 | Australia | Intervention study |
| 22 | **Gibbs KA.** Teaching Student Nurses to be Culturally Safe: Can It Be Done? Journal of Transcultural Nursing 16(4): 356-360. | 2005 | New Zealand | Perspective/viewpoint article |
| 23 | **Govere L, Govere EM.** How Effective is Cultural Competence Training of Healthcare Providers on Improving Patient Satisfaction of Minority Groups? A Systematic Review of Literature. Worldviews on Evidence-Based Nursing 13(6): 402-410. | 2016 | USA | Systematic review, 7 studies included. |
| 24 | **Grote E.** Principles and Practices of Cultural Competency: A Review of the Literature. Canberra: Indigenous Higher Education Advisory Council (IHEAC), Australian Government, Department of Education Employment and Workplace Relations. | 2008 | Australia | Literature review & key informant interviews |
| 25 | **Hall M, Guidry J.** Literature Review of Cultural Competence Curriculum within the United States: An Ethical Implication in Academic Preparational Programs. Education in Medicine 5(1): e6-e13. | 2013 | USA | Literature review (16 articles) |
| 26 | **Healey P, et al.** Cultural adaptations to augment health and mental health services: a systematic review. BMC Health Services Research 17. | 2017 | Canada | Systematic review (69 reports and studies) |
| 27 | **Hook JN, et al.** Cultural Humility in Psychotherapy Supervision. American Journal of Psychotherapy 70(2): 149-166. | 2016 | USA | Primary research |
| 28 | **Hook JN, et al.** Cultural humility: Measuring openness to culturally diverse clients. Journal of Counseling Psychology 60(3): 353-366. | 2013 | USA | Primary research |
| 29 | **Horvat L, Horey D, Romios P, Kis-Rigo J.**Cultural competence education for health professionals. Cochrane Database of Systematic Reviews 2014, Issue 5. Art. No.: CD009405. DOI: 10.1002/14651858.CD009405.pub2. | 2014 | Australia (Cochrane Review) | Systematic review |
| 30 | **Kirmayer LJ.** Rethinking cultural competence. Transcultural Psychiatry 49(2): 149-164. | 2012 | USA | Editorial |
| 31 | **Kumagai A, Lypson M.** Beyond cultural competence: Critical consciousness, social justice, and multicultural education Academic Medicine 84(6): 782-787. | 2009 | USA | Perspective/viewpoint article |
| 32 | **Laverty M, et al.** Embedding cultural safety in Australia’s main health care standards. Medical Journal of Australia 207(1): 15-16. | 2017 | Australia | Perspective/viewpoint article |
| 33 | **Leininger M.** Culture Care Theory: A Major Contribution to Advance Transcultural Nursing Knowledge and Practices. Journal of Transcultural Nursing 13(3): 189-192. | 2002 | USA | Descriptive review |
| 34 | **Lin CJ, et al.** Cultural competence of healthcare providers: A systematic review of assessment instruments. Journal of Nursing Research 25(3): 174-186. | 2017 | Taiwan | Systematic review (57 articles) |
| 35 | **Maier-Lorentz MM.** Transcultural nursing: its importance in nursing practice. Journal of Cultural Diversity 15(1): 37-43. | 2008 | USA | Perspective/viewpoint article |
| 36 | **Main C, et al.** Cultural safety and cultural competence: what does this mean for physiotherapists? New Zealand Journal of Physiotherapy 34(3): 160-166. | 2006 | New Zealand | Perspective/viewpoint article |
| 37 | **Malat J.** The appeal and problems of a cultural competence approach to reducing racial disparities. Journal of General Internal Medicine 28(5): 605-607. | 2013 | USA | Editorial |
| 38 | **McGough S, et al.** Experience of providing cultural safety in mental health to aboriginal patients: A grounded theory study. International Journal of Mental Health Nursing 27(1): 204-213. | 2018 | Australia | Primary qualitative research |
| 39 | **McLennan V, et al.** Creating Culturally Safe Vocational Rehabilitation Services for Indigenous Australians: A Brief Review of the Literature. The Australian Journal of Rehabilitation Counselling 22(2): 93-103. | 2016 | Australia | Literature review (vocational rehab) |
| 40 | **Miller S.** Cultural humility is the first step to becoming global care providers. JOGNN - Journal of Obstetric, Gynecologic, and Neonatal Nursing 38(1): 92-93. | 2009 | USA | Editorial |
| 41 | **Milne T, et al.** Development of the Awareness of Cultural Safety Scale: A pilot study with midwifery and nursing academics. Nurse Education Today 44(Supplement C): 20-25. | 2016 | Australia | Primary research |
| 42 | **Papps, E. Ramsden, I.** Cultural safety in nursing: the New Zealand experience. International Journal for Quality in Health Care 8(5): 491-497. | 1996 | New Zealand | Descriptive review |
| 43 | **Phiri J, et al.** Cultural safety and its importance for Australian midwifery practice. Collegian 17(3): 105-111. | 2010 | Australia | Perspective/viewpoint article |
| 44 | **Polaschek NR.** Cultural safety: a new concept in nursing people of different ethnicities. Journal of Advanced Nursing 27(3): 452-457. | 1998 | New Zealand | Descriptive review |
| 45 | **Price EG, et al.** A systematic review of the methodological rigor of studies evaluating cultural competence training of health professionals. Academic Medicine 80(6): 578-586. | 2005 | USA | Systematic review |
| 46 | **Ramsden I.** Cultural Safety and Nursing Education in Aotearoa and Te Waipounamu. A thesis submitted to the Victoria University of Wellington in fulfilment of the requirements for the degree of Doctor of Philosophy in Nursing. Department of Nursing. Wellington, Victoria University of Wellington Doctor of Philosophy in Nursing: 211. | 2002 | New Zealand | Thesis |
| 47 | **Ratima M, et al.** Cultural competence for physiotherapists: reducing inequalities in health between Maori and non-Maori. New Zealand Journal of Physiotherapy 34(3): 153-159. | 2006 | New Zealand | Perspective/viewpoint article |
| 48 | **Richardson A, et al.** Expressions of cultural safety in public health nursing practice. Nursing Inquiry 24(1). | 2017 | New Zealand | Primary qualitative research |
| 49 | **Richardson S.** Aoteaoroa/New Zealand nursing: from eugenics to cultural safety. Nursing Inquiry 11(1): 35-42. | 2004 | New Zealand | Review article |
| 50 | **Ringer J.** Cultural safety and engagement: Keys to improving access to care. Healthcare Management Forum 30(4): 213-217. | 2017 | USA | Descriptive article |
| 51 | **Rowan MS, et al.** Cultural competence and cultural safety in Canadian Schools of Nursing: A mixed methods study. International Journal of Nursing Education Scholarship 10(1). | 2013 | Canada | Research article |
| 52 | **Shen Z.** Cultural Competence Models and Cultural Competence Assessment Instruments in Nursing: A Literature Review. Journal of Transcultural Nursing 26(3): 308-321. | 2015 | USA | Descriptive review |
| 53 | **Smye V, Browne A.** 'Cultural safety' and the analysis of health policy affecting aboriginal people. Nurse Researcher (through 2013) 9(3): 42-56. | 2002 | Canada | Descriptive article |
| 54 | **Tervalon M, Murray-Garcia J.** Cultural humility versus cultural competence: a critical distinction in defining physician training outcomes in multicultural education. Journal of Health Care for the Poor & Underserved 9(2): 117-125. | 1998 | USA | Perspective/viewpoint article |
| 55 | **Truong M, et al.** Interventions to improve cultural competency in healthcare: a systematic review of reviews. BMC Health Services Research 14: 99. | 2014 | Australia | Systematic review (19 articles) |
| 56 | **Watt K, et al.** Developing cultural competence in general practitioners: an integrative review of the literature. BMC Family Practice 17. | 2016 | Australia | Systematic literature review (50 articles) |
| 57 | **Wepa D.** An exploration of the experiences of cultural safety educators in New Zealand: an action research approach. Journal of Transcultural Nursing 14(4): 339-348. | 2003 | New Zealand | Primary qualitative research |
| 58 | **Whaley AL.** Cultural sensitivity and cultural competence: toward clarity of definitions in cross-cultural counselling and psychotherapy. Counselling Psychology Quarterly 21(3): 215-222. | 2008 | USA | Literature search, psychology focused. |
| 59 | **Wilson D, Neville S.** Culturally safe research with vulnerable populations. Contemporary Nurse 33(1): 69-79. | 2009 | New Zealand | Descriptive review |
